# Optimization of radiation protection using diagnostic reference values: current approaches and future directions

**DOI:** 10.1093/rpd/ncaf106

**Published:** 2026-03-13

**Authors:** Anja Almén

**Affiliations:** Medical Radiation Physics, Department of Translational Medicine, Lund University, Inga Marie Nilssons gata 47, SE-205 02 Malmö, Sweden; Swedish Radiation Safety Authority, Solna Strandväg 96, SE-171 16, Stockholm, Sweden

## Abstract

Diagnostic reference levels (DRLs) were introduced as a tool to optimize radiation protection in medical imaging. Conditions for dose assessment have evolved significantly. This study explores how effectively the current DRL system functions and whether it requires adjustment. The analysis focuses on the national DRL system in Sweden and includes European projects and guidelines for reference. The variability in typical doses has decreased. National DRL values have been lowered; however, values in nuclear medicine have seen minimal change. These developments present challenges in revising the values. The increased availability of digital data offers both opportunities and challenges. The DRL system may include specific indications, larger patient cohorts, and consideration of patient size. To remain relevant, the DRL system must be updated to reflect changes in clinical practice. The integration of image quality assessment into the DRL framework is one critical area that requires further development.

## Introduction

The principle of optimization in radiological protection was introduced by the International Commission on Radiological Protection (ICRP) in 1977 [1] and extends to also encompass medical exposures, including diagnostic imaging. In medical contexts, optimization of radiological protection, alongside justification of exposure, constitutes a fundamental component of the protection framework [2], particularly as dose limits do not apply to individuals undergoing medical exposure.

The most recent formulation of the optimization principle within the ICRP’s system of radiological protection is presented in ICRP Publication 103 [2] and ICRP Publication 105 [3] and the process of optimization has been further discussed in ICRP Publication 154 [4]. Nevertheless, these principles are presented at a high level of abstraction, which can pose challenges for implementation in routine clinical practice and thus meet the goal set out by the ICRP [3]: “…management of the radiation dose to the patient to be commensurate with the medical purpose…” (ICRP 105, paragraph 70 page 33).

Initial dose surveys revealed substantial variability in radiation doses between different healthcare facilities and often revealed positively skewed dose distributions [e.g. 5], where high-dose outliers could be identified. The variations may stem from differences in equipment, imaging protocols, or clinical practices, However, it is often unclear whether such observational studies—frequently conducted in research settings—lead to meaningful investigation or action in the clinical environment. Furthermore, without comparative data from other facilities, it can be difficult for a clinic to interpret the significance of its dose values.

The concept of diagnostic reference levels (DRLs) was introduced in 1996 [6] as a practical tool to support the optimization principle in medical imaging, and it has been further developed in ICRP Publication 135 [7]. The overarching goal of employing reference levels in medical imaging is to promote optimization in clinical practice, but also more specifically to indicate when a radiological protection action may be warranted. The DRL system is designed to identify procedures that result in unusually high patient doses by providing a reference value against which individual clinics can compare their typical values. A prerequisite is to define a typical dose for a standard examination, for a standard-sized patient to be used as a benchmark and some challenges were early defined [8]. The integration of the DRL concept into the optimization process has been explored in previous research and found to be well-aligned with its objectives [9].

The DRL concept was incorporated into radiation protection standards issued by both the International Atomic Energy Agency (IAEA) [10] and the European Commission (EC) [11]. However, a critical question emerges: do current DRL strategies remain well-suited to the evolving clinical, technological, and methodological landscape of modern medical imaging?

This study aims to explore the evolving requirements of the DRL system in contemporary healthcare. The specific objectives are to: (i) assess the application of DRLs in the past and present; (ii) analyze historical and current typical dose data; (iii) identify potential factors influencing DRL implementation; and (iv) propose modifications to enhance the effectiveness of the DRL system in the future.

The study is based on over two decades of experience with the DRL system in Sweden, based on national data and practical insights from its application.

## Methods

### Application of DRLs in historical and contemporary contexts

European guidelines and documentation from various EU projects [12–14], alongside Swedish standards [15, 16] and national reports [17], were utilized to investigate trends in the application of DRLs. Particular emphasis was placed on the implementation and evolution of the DRL system in Sweden. A comparative analysis was conducted of national diagnostic reference levels (NDRLs) specified in the 2002 and 2023 regulatory frameworks [15, 16], focusing on selected X-ray and nuclear medicine examinations. Only examinations that remained consistently defined across both regulatory periods were included in the comparison of NDRL values to ensure validity.

### Evolution of typical doses and dose distributions

Radiation dose data from a nationwide Swedish survey of data collected around the year 2005 [17] were analyzed together with contemporary data on typical doses and dose distributions for conventional lung radiography, mammography screening, and thoracic computed tomography (CT) collected in the time period 2023 to 2025 by the Swedish Radiation Safety Authority (SSM). The objective was to assess the development of radiation exposure levels. In recent regulatory updates, examinations have been further stratified by clinical indication. Nonetheless, the procedures included in this analysis are considered to be largely standardized. DRL values for mammography screening are reported per exposure, whilst CT examinations were evaluated using the volume CT dose index (CTDIvol). The broad nature of the clinical indications [16] enhances the robustness of the comparison, minimizing potential bias introduced by changes in clinical indications over time. Activity distribution for reported typical values for bone scintigraphy was also analyzed for the years 2002 and 2023. Additionally, reported values for administered activity per body weight were studied for two hospitals for positron emission tomography (PET) oncology examinations reported in 2020 and 2023, and in 2021 and 2024, respectively.

### Prospective factors affecting future implementation of DRLs

Contemporary trends in medical imaging were reviewed to identify potential factors that may influence the future application of the DRL framework. These considerations were informed by the aforementioned European studies and other publications and have the ambition to reflect emerging practices and technological developments within the field. This resulted in a discussion about potential adjustments of the DRL system to modern health care.

## Results

### The application of DRL in the past and present

The European guidelines on DRLs were issued in 1999 [12]. These guidelines provided both general information on the purpose of the DRL system and more detailed recommendations, such as appropriate radiation quantities for various imaging modalities. Whilst the guidelines acknowledged that patient examinations could serve as the basis for determining typical doses, using physical phantoms was not excluded. A range of diagnostic radio and nuclear medicine procedures were included, accompanied by suggested DRL values. The guidelines emphasized the importance of accounting for patient size, recommending that typical dose values be derived from a group of at least 10 patients with a mean weight of 70 ± 3 kg. The need to extend the system to more complex procedures—such as CT, image-guided interventions, and paediatric imaging—was also highlighted. However, the inclusion of these examinations was acknowledged as more technically and logistically challenging.

A key distinction was noted between diagnostic radiology and nuclear medicine: in nuclear medicine, DRLs function as target values rather than a value that trigger investigation. Despite this, it was found that activity levels used across Europe for the same radiopharmaceutical and examination exhibited substantial variability, indicating that DRL might be useful also for nuclear medicine. Image quality and clinical outcomes were also discussed in the context of DRLs. The digitalization of radiology was recognized as a significant factor impacting dose levels. Digital detectors were introduced with essentially no intrinsic upper dose limit that would render the images unusable, potentially allowing higher radiation doses to persist undetected, indicating a benefit of applying DRLs.

In 2018, a European study [13] focused specifically on paediatric diagnostic imaging was conducted. The report recommended the use of measurable dose quantities obtained directly from imaging equipment and suggested grouping patients by weight (for body examinations) or age (for head examinations). It emphasized that DRL values should be based on patient data and should be updated regularly—ideally every 3–5 y. The report did not advise on new European DRL values. The study is based on over two decades of experience with the DRL system in Sweden, based on national data and practical insights from its application. The report also mentioned the DRL curves, a concept that had been previously suggested [e.g. 18]. Such curves may also reveal the degree of adaption of the radiation exposure to different body habitus. Different studies including a study in the Nordic countries [19] explored the use of paediatric DRLs curves.

In 2021, another European project [14] focusing primarily on adult diagnostic imaging examinations was conducted. The application of clinical indication-based DRLs gained traction. The report identified inconsistent terminology, reporting formats, and consistent classification of clinical indications as important. The report did advise on new European DRL values for a number of procedures.

In Sweden, national studies on typical radiation doses have been conducted since the early 2000s, and NDRLs were first established in 2002 based on the results of these studies. The structure and methodology of the Swedish system follow international recommendations, with a focus on standardized data collection. There were more substantial updates of the NDRLs in 2018 and 2023. Particular attention has been given to paediatric examinations. A Nordic study subsequently derived DRL values based on patient weight groups for several paediatric procedures [20] and data from this study was used to set NDRLs. For nuclear medicine procedures, including hybrid imaging modalities such as PET/CT, weight-based administered activity in line with ICRP recommendations [8] was included in the latest update of NDLRs. The importance of incorporating clinical indications defining standard examinations was also followed. The latest update in 2023, includes NDRL values for a number of examinations, Table 1 presents values for selected examinations included in 2002 and 2023. In general, most NDRL values have decreased substantially over time, with the exceptions of mammography screening and nuclear medicine examinations, where changes have been limited.

**Table 1:** NDRLs in Sweden, for a selected number of diagnostic radiology and nuclear medicine procedures for the year 2002 and 2023 [15, 16].

Examination	NDRL 2002	NDRL 2023
Conventional lung exam, P_KA_ (all views)	0.6 Gycm^2^	0.25 Gycm^2^
Conventional lumbar spine, P_KA_ (all views)	10 Gycm^2^	4.0 Gycm^2^
Mammography screening (per view)	1.3 mGy	1.1 mGy
Coronary angiography	80 Gycm^2^	11 Gycm^2^
CT brain, CTDIvol (total DLP)	75 mGy /1200 mGycm	46 mGy /850 mGycm
CT thorax, CTDIvol (total DLP)	20 mGy /600 mGycm	6.1 mGy /245 mGycm
NM bone scintigraphy ^99m^ Tc-MDO/HDP	600 MBq	7.5 MBq/kg (525 MBq)
NM renal scintigraphy ^99m^ Tc-MAG3	110 MBq	1.5 MBq/kg (105 MBq)
NM lung perfusion ^99m^ Tc-MAA	125 MBq	2 MBq/kg (140 MBq)
NM myocard scintigraphy ^99m^ Tc-tetrofosmin	600 MBq	7.5 MBq/kg (525 MBq)
NM PET oncological procedures ^18^F-FDG	350 MBq	3.5 MBq/kg (245 MBq)

### Trends in typical doses and dose distributions

**Figure 1:**
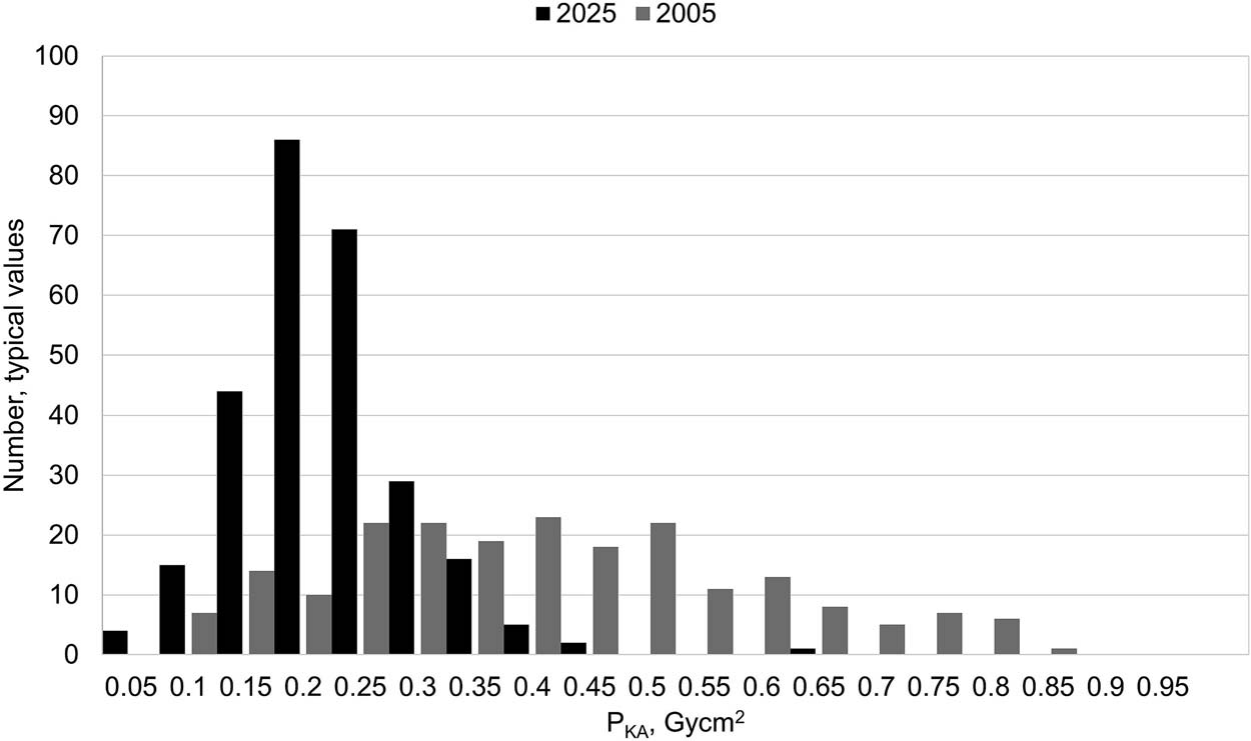
Distribution of reported typical patient doses (P_KA_, Gy·cm^2^) for conventional lung examinations in 2005 and 2025, collected in national surveys.

**Figure 2:**
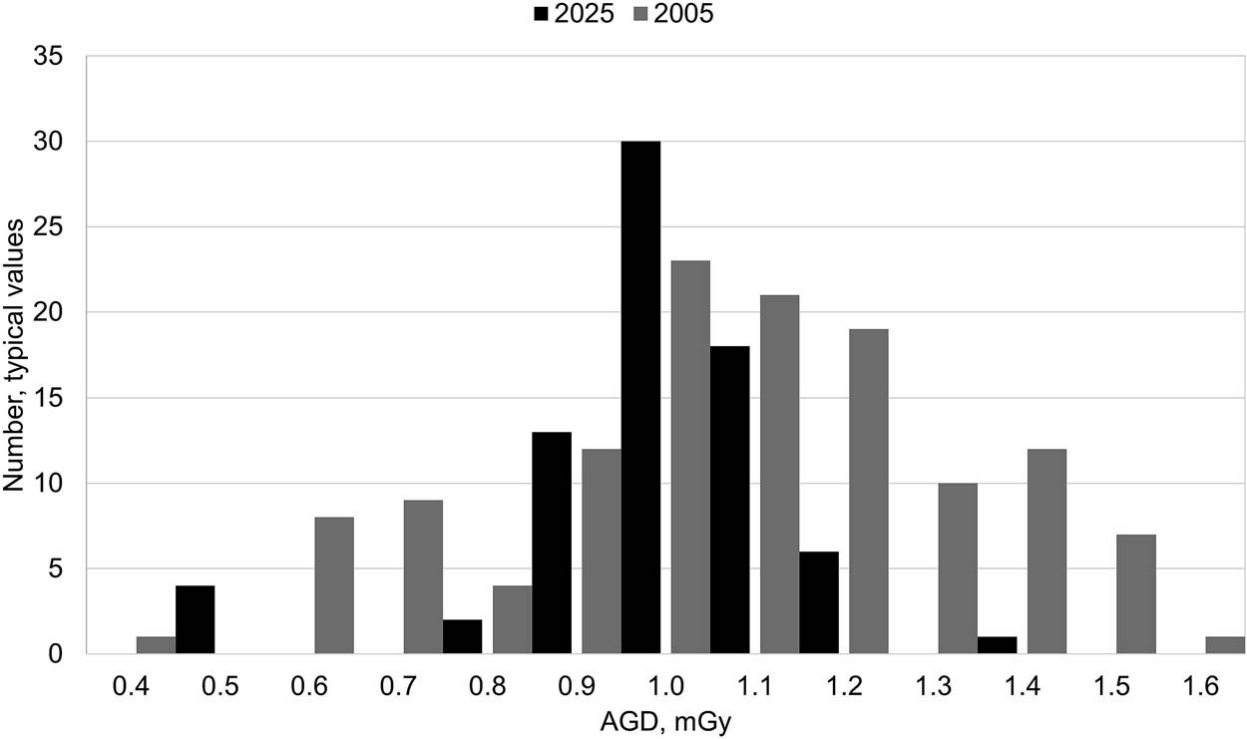
Distribution of reported typical average glandular dose (mGy) for mammography screening, one view, in 2005 and 2025, collected in national surveys.

**Figure 3:**
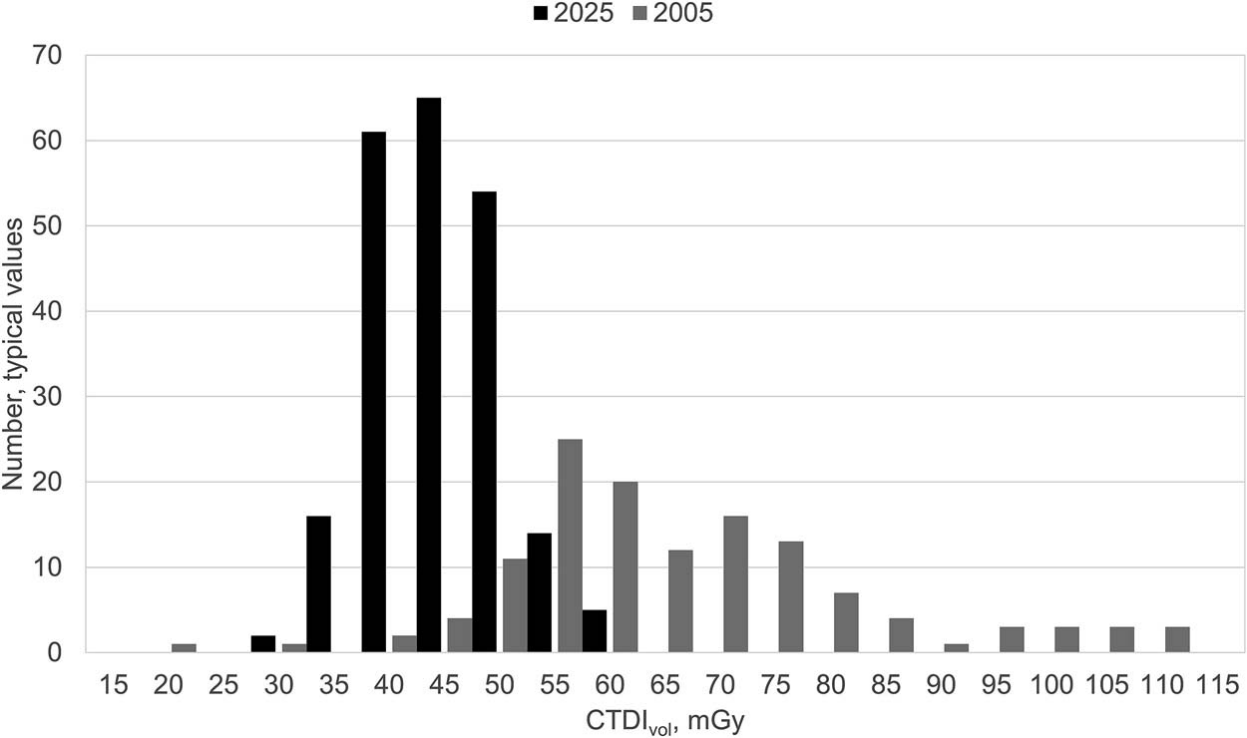
Distribution of reported typical doses, CTDI_vol_ (mGy) for CT examination of brain without contrast, in 2005 and 2025, collected in national surveys.

Figures 1–3 show the dose distributions for conventional lung radiography, mammography screening and CT of the brain respectively. For lung radiography, the median typical dose has decreased from 0.39 Gycm^2^ to 0.19 Gycm^2^, and the overall dispersion has narrowed. It is noteworthy that even in 2005, many reported typical doses fell within the same range as current values. In mammography screening, the median average glandular dose, 1.0 mGy, has remained stable, but the distribution has become more concentrated around the median. The reported values for CTDI_vol_ regarding CT brain also showed that the overall dispersion has narrowed and the median values lowered from 60 mGy to 42 mGy. These observations are in line with the primary objective of DRLs—to reduce the number of values in the upper end of dose distributions and optimize patient protection. Similar trends may be expected for other types of X-ray examinations, particularly in light of the lowered NDRLs observed in recent updates.

**Figure 4:**
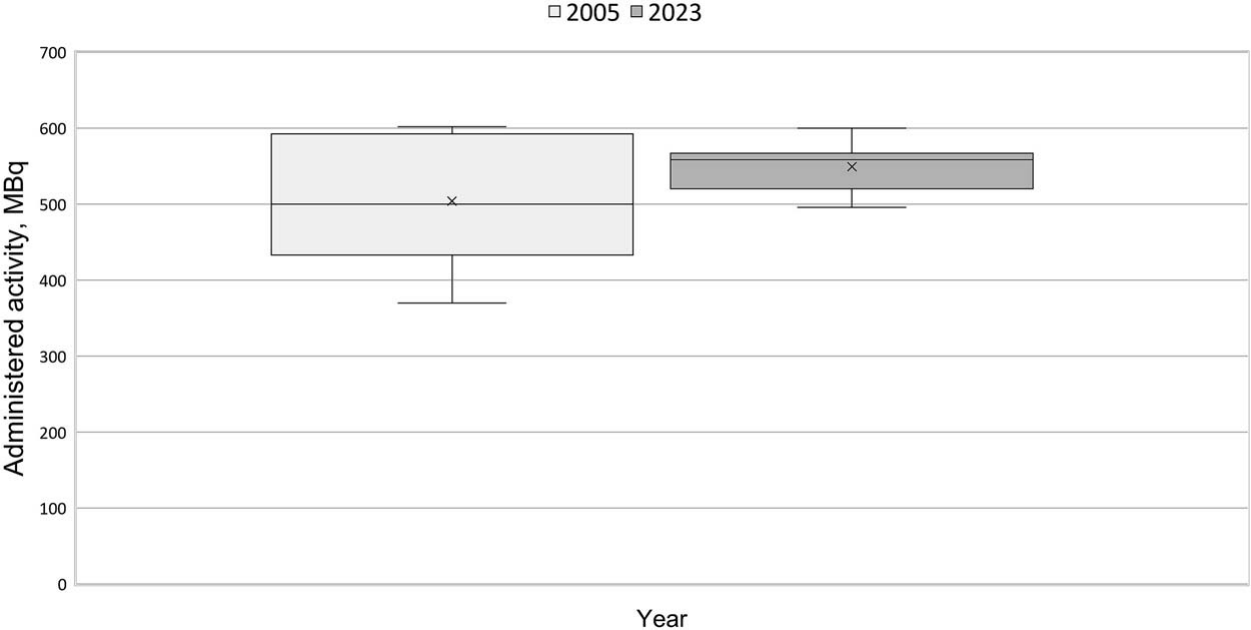
Distribution of reported typical doses, average administered activity (MBq), for bone scintigraphy, in 2005 and 2023, collected in national surveys.

**Figure 5:**
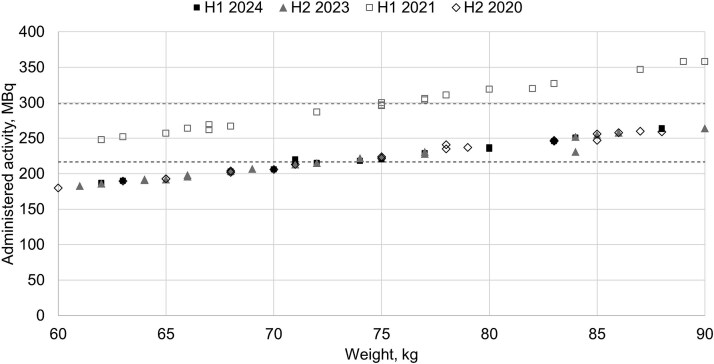
Reported typical doses, administered activity per body weight. MBq/kg, oncological procedures ^18^F-FDG for two hospitals H1 and H2. Dashed lines indicate median activity per body weight for H1. Data collected in national surveys.

In contrast, the administered activity in nuclear medicine has remained relatively unchanged for over 20 y [21]. Differences in typical doses across facilities are not substantial. However, the quantity used for DRL has shifted to administered activity per kilogramme of body weight for many of the procedures. This assumes that activity is consistently adjusted for patient weight, which is not always the case in clinical practice. Figure 4 shows the distribution of administered activity for bone scintigraphy also showing a narrowing of the overall dispersion but not a lowered mean administered activity. Furthermore, about 70% of the hospitals do not adjust administered activity according to body weight. However, newer radiopharmaceuticals, used in PET imaging, that have been incorporated into the DRL system, show a larger percentage adjustment for body weight. Figure 5 shows data from two hospitals for PET oncology examinations, and administered activities per body weight reported in 2020 and 2023, and 2021 and 2024, respectively. Both hospitals demonstrated consistent application of weight-based administration. One facility reported a reduction in administered activity of ⁓25%, whilst the other reported identical values across both years. All other hospitals also reported administered activity per body weight for this particular examination.

## Discussion

The DRL system has been found useful for raising awareness about patient doses and initiating dose surveys in the clinical domain. This section will discuss potential factors influencing the application of the future DRL system.

### Availability of dose data and other clinical data

The establishment and clinical use of NDRLs rely on dose data collection and national dose surveys. In the past—and even today—collecting large amounts of patient data can be cumbersome. Typically, data from a smaller number of patients, ⁓20 defined by body weight, has been collected for each modality and anatomical region. Modern imaging systems now provide dose data, supported by dose management systems, enabling the collection of much larger datasets [22]. However, other important information, such as body habitus and clinical indication, may not be included.

In the context of DRLs, it is essential to link specific protocols to clinical indications, along with other relevant data from hospital information systems. It is not self-evident that all necessary data is immediately available. Nevertheless, there appears to be increasing potential to utilize data more effectively—allowing for the collection of data from better-defined and larger cohorts, enabling faster data acquisition and, consequently, more timely updates of NDRLs.

### The distribution of doses

A trend towards more evenly distributed typical doses can be observed. This, combined with the fact that all typical doses carry uncertainties in their determination, presents a challenge to the DRL system. In the future, smaller shifts in typical doses—both increases and decreases—may be observed as a result of this uncertainty rather than actual changes in patient doses.

Defining a reference level as the 75th percentile may no longer be sufficiently effective, and further changes to NDRLs may not be expected. The reference level may function more as a gatekeeper than as a driver of radiation protection actions. Nevertheless, it can still serve as a gatekeeper in a similar manner as seen in nuclear medicine.

### Demand for more personalized data

Over time, there has been a growing demand for more precisely defined procedures based on clinical indication rather than anatomical region, with the aim of capturing a more specific patient cohort. As a result, it may also be expected that the influence of different patient characteristics should be incorporated into the DRL system. This could include a radiation dose quantity that more accurately reflects the absorbed dose to the body. Such quantities have already been defined for CT, such as the size-specific dose estimate, and the use of water-equivalent diameter to define body size in the context of CT [23, 24]. Furthermore, it has been highlighted that there is a need to include factors that properly account for adjustments in radiation dose based on different patient habitus.

As mentioned in the context of nuclear medicine, administered activity per body weight has been integrated into the system. For paediatric examinations, both weight groups and DRL curves supporting typical doses have been introduced. The rationale for extending this approach to adult diagnostic radiology is evident—that is, to introduce weight groups or DRL curves for adult X-ray diagnostics as well.

### Clinical image quality

Throughout this period, the importance of including image quality in connection with DRLs has been highlighted and was also addressed in the latest ICRP guidance [8]. Unfortunately, the criteria for and evaluation of image quality in this context have been poorly defined. Over time, the conditions for dose assessment have evolved significantly, and the ability to evaluate medical information through imaging technology and information technology (IT) capabilities has increased tremendously over the past decade. This includes the use of automated artificial intelligence (AI) systems, and recent studies suggest that such systems may be developed for this purpose [25]. Quality-related challenges have shifted from purely measurement issues to also include concerns about data transmission and database content. However, clinical image quality criteria—such as those included in the European guidelines [26–28]—need to be revised and actively applied in the clinical context.

## Conclusions

The DRL system has played an important role in raising awareness of patient radiation doses and promoting the measurement and analysis of radiation exposure in clinical practice. DRLs may have significantly influenced dose levels. Over time, the variation in typical dose distributions for several X-ray examinations has decreased, making it more challenging to revise NDRL values and maintain the system’s timeliness. To enhance its relevance, there is a need to incorporate clinically based image quality parameters into the DRL framework. The DRL system should also leverage the large volume of available medical data—including radiation dose metrics—to support dose adjustments based on patient body habitus, preferably whilst also accounting for image quality.
